# Evaluation of the Panbio COVID-19 Antigen Rapid Diagnostic Test in Subjects Infected with Omicron Using Different Specimens

**DOI:** 10.1128/spectrum.01250-22

**Published:** 2022-06-02

**Authors:** Rafael Mello Galliez, Larissa Bomfim, Diana Mariani, Isabela de Carvalho Leitão, Anna Carla Pinto Castiñeiras, Cassia Cristina Alves Gonçalves, Bianca Ortiz da Silva, Pedro Henrique Cardoso, Monica Barcelos Arruda, Patricia Alvarez, Rodrigo Brindeiro, Victor Akira Ota, Débora Gomes Marins Rodrigues, Luciana Jesus da Costa, Orlando da Costa Ferreira, Terezinha Marta Pereira Pinto Castiñeiras, Debora Souza Faffe, Amilcar Tanuri

**Affiliations:** a Núcleo de Enfrentamento e Estudos de Doenças Infecciosas Emergentes e Reemergentes, Universidade Federal do Rio de Janeiro, Rio de Janeiro, Brazil; b Laboratório de Virologia Molecular, Departamento de Genética, Instituto de Biologia, Universidade Federal do Rio de Janeiro, Rio de Janeiro, Brazil; c Instituto de Biofísica Carlos Chagas Filho, Universidade Federal do Rio de Janeiro, Rio de Janeiro, Brazil; d Instituto de Microbiologia Paulo de Góes, Universidade Federal do Rio de Janeiro, Rio de Janeiro, Brazil; e Departamento de Doenças Infecciosas e Parasitárias, Faculdade de Medicina, Universidade Federal do Rio de Janeiro, Rio de Janeiro, Brazil; f Hospital Universitário Clementino Fraga Filho, Centro de Ciências da Saúde, Universidade Federal do Rio de Janeiro, Rio de Janeiro, Brazil; g Decania, Centro de Ciências da Saúde, Universidade Federal do Rio de Janeiro, Rio de Janeiro, Brazil; h Instituto de Tecnologia de Imunobiológicos Bio-manguinhos, Fundação Oswaldo Cruz/Fiocruz, Rio de Janeiro, Brazil; Hôpital Saint-Louis

**Keywords:** Ag-RDT, Omicron, rapid test

## Abstract

Community testing is a crucial tool for the early identification of severe acute respiratory syndrome coronavirus 2 (SARS-CoV-2) infection and transmission control. The emergence of the highly mutated Omicron variant (B.1.1.529) raised concerns about its primary site of replication, impacting sample collection and its detectability by rapid antigen tests. We tested the performance of the Panbio antigen rapid diagnostic test (Ag-RDT) using nasal and oral specimens for COVID-19 diagnosis in 192 symptomatic individuals, with quantitative reverse transcription-PCR (RT-qPCR) of nasopharyngeal samples as a control. Variant of concern (VOC) investigation was performed with the 4Plex SARS-CoV-2 screening kit. The SARS-CoV-2 positivity rate was 66.2%, with 99% of the positive samples showing an amplification profile consistent with that of the Omicron variant. Nasal Ag-RDT showed higher sensitivity (89%) than oral (12.6%) Ag-RDT. Our data showed good performance of the Ag-RDT in a pandemic scenario dominated by the Omicron VOC. Furthermore, our data also demonstrated that the Panbio COVID-19 antigen rapid diagnostic test does not provide good sensitivity with oral swabs for Omicron Ag-RDT detection.

**IMPORTANCE** This study showed that the antigen rapid test for COVID19 worked fine using nasal swabs when it was utilized in patients infected with the Omicron variant, showing a concordance with PCR in 93% of patients tested. The nasal swab yielded more reliable results than the oral swab when an antigen rapid diagnosis test (the Panbio COVID-19 antigen rapid diagnostic test) was used in patients infected with the Omicron variant.

## INTRODUCTION

Since the beginning of the COVID-19 pandemic, the World Health Organization has advised widespread testing to identify infected individuals and control the onward transmission of the virus (1, 2). Severe acute respiratory syndrome coronavirus 2 (SARS-CoV-2) can spread quickly in the global population, and new variants can emerge due to different selective pressures (3). In fact, the prolonged circulation of the virus has resulted in the emergence of multiple variants in many countries during the COVID-19 pandemic. Some variants are of great interest to public health due to their critical mutations in the spike (S) protein. These mutations, named variants of concern (VOCs), can change the binding of neutralizing antibodies as well as the affinity of S to the angiotensin-converting enzyme 2 (ACE2) receptor (4, 5). Since June 2021, we have faced several global waves of VOCs, such as Alpha, Beta, Gamma, Delta, and, more recently, Omicron (5, 6).

The latest Omicron variant (B.1.1.529) quickly replaced the Delta variant and recently dominated the pandemic (7). Omicron is a highly mutated strain, including 50 mutations in its genome and at least 32 in the spike protein. The following mutations are present in Omicron spike protein: A67V, Δ69–70, T95I, G142D, Δ143–145, Δ211, L212I, insertion 214-EPE, G339D, S371L, S373P, S375F, K417N, N440K, G446S, S477N, T478K, E484A, Q493R, G496S, Q498R, N501Y, Y505H, T547K, D614G, H655Y, N679K, P681H, N764K, D796Y, N856K, Q954H, N969K, and L981F. These variations could impact Omicron's ability to escape from monoclonal antibodies and from neutralizing antibodies elicited by COVID-19 vaccines (7, 8). Indeed, studies have reported approximately 25-fold to 40-fold reductions in serum neutralizing activity compared to historical D614G-containing strains from individuals immunized with the Pfizer BNT162b2 and AstraZeneca AZD1222 vaccines (9–13).

Quantitative reverse transcription-PCR (RT-qPCR) is considered the “gold standard” test in COVID-19 diagnosis to detect the viral genetic material in different body fluids (14). SARS-CoV-2 starts its replication in the upper respiratory compartment, making the nasopharynx the most informative site for swab collection since it is rich in viral RNA and antigens (15). However, nasal swabs have also shown good sensitivity (16). Other locations and fluids can also be used in COVID-19 molecular tests, such as oral or gingival swabs and spit saliva.

Whereas RT-qPCR plays an essential role in detecting infected individuals, antigen rapid diagnostic tests (Ag-RDTs) arose as a tool for rapid SARS-CoV-2 viral protein detection in a less expensive way (1). Most Ag-RDTs use a monoclonal antibody directed against the nucleocapsid (N) protein. Mutations in VOCs are frequently present in the S protein. However, they can also occur at nonstructural and other structural proteins such as N or open reading frames (ORFs) 3, 6, 7, and 8 (4, 5). The principal concern is that VOC-related mutations can disturb the binding of the N capture monoclonal antibodies, decreasing test sensitivity. Omicron has a unique mutation in N protein (P13L) and a deletion of two amino acids (ER) in positions 32 and 33 (6). The impact of these N protein mutations on the binding of monoclonal antibodies responsible for the capture and Ag-RDT development is unknown. The other way VOCs can impact the Ag-RDT results is by changing the initial site of viral replication. Usually, swab specimens are collected from the nasopharynx or nasal cavity. However, if VOC viral replication initiates in the oral cavity, before spreading to the nasopharynx and nasal cavity, the usual collection could fail to detect the initial phase of infection. This fact was reported by Marais et al. in 2021 during the beginning of Omicron spread in South Africa (17). The authors showed a better sensitivity using oral swabs than nasal ones. In order to explore the SARS-CoV-2 antigen detection in different body fluids/compartments in symptomatic patients infected with the Omicron variant, we analyzed the diagnostic performance of nasal and oral swabs using the Panbio COVID-19 Ag test device during acute infection. We also determined the persistence/disappearance of Ag-RDT positivity in the different specimens until the 7th day after a positive diagnosis by RT-qPCR.

## RESULTS

From 17 January 2022 to 7 February 2022, a total of 192 individuals were tested for SARS-CoV-2 by RT-qPCR and Panbio Ag-RDT simultaneously at the Center for COVID-19 Diagnosis of the Federal University of Rio de Janeiro. Table 1 describes the general characteristics of the study cohort. Females represented the majority of patients (65.6%), with a mean age of 39 years. Most individuals were tested within 3 days of symptom onset (66.1%). Nearly all patients sampled were fully immunized against COVID-19 (97.9%), 67.2% with a 3rd vaccine shot.

**TABLE 1:** Cohort general characteristics

Characteristic	Result for characteristic
No. of patients	192
Age in yr, avg (range)	39 (21–74)
Gender, no. (%)	
Female	126 (65.6)
Male	66 (34.4)
DSSO, mode (range)^*a*^	3 (0–7)
≤3	127 (66.1)
4–7	65 (33.9)
Vaccination, no. (%)	
1 dose	4 (2.1)
2 doses	59 (30.7)
≥3 doses	129 (67.2)
Follow-up, no. (%)	32 (16.7)
Follow-up DSSO, mode (range)	10 (2–12)
Positive results, no. (%)	
RT-qPCR	127 (66.2)
Ag_Nasal	113 (58.9)
Ag_Oral	16 (8.6)

a DSSO, days since symptom onset.

The SARS-CoV-2 positivity rate in our cohort was higher using RT-qPCR of nasopharyngeal swabs (66.2%) than Ag-RDTs. Nasal specimens yielded higher positivity rate (58.9%) than oral (8.6%) Ag-RDT (Table 1). The performance of Ag-RDT was higher when using nasal specimens than oral specimens, yielding 89% sensitivity, compared with 12.6% in the oral counterpart. The specificity of Ag-RDT was 100%, regardless of the specimen tested (Table 2).

**TABLE 2:** Sensitivity and specificity of Ag-RDT in relation to SARS-CoV-2 RT-qPCR result

Test result	No. with RT-qPCR result			% sensitivity (95% CI)	% specificity (95% CI)	% Positive predictive value (95% CI)	% Negative predictive value (95% CI)
	Positive	Negative	Total				
Ag-RDT							
Nasal							
Positive	113	0	113	89.0 (82.4–93.3)	100.0 (94.4–100.0)	100.0 (96.7–100.0)	82.3 (72.4–89.1)
Negative	14	65	79				
Total	127	65	192				

Oral							
Positive	16	0	16	12.6 (7.9–19.5)	100.0 (94.4–100.0)	100.0 (80.6–100.0)	36.9 (30.2–44.3)
Negative	111	65	176				
Total	127	65	192				

**FIG 1:**
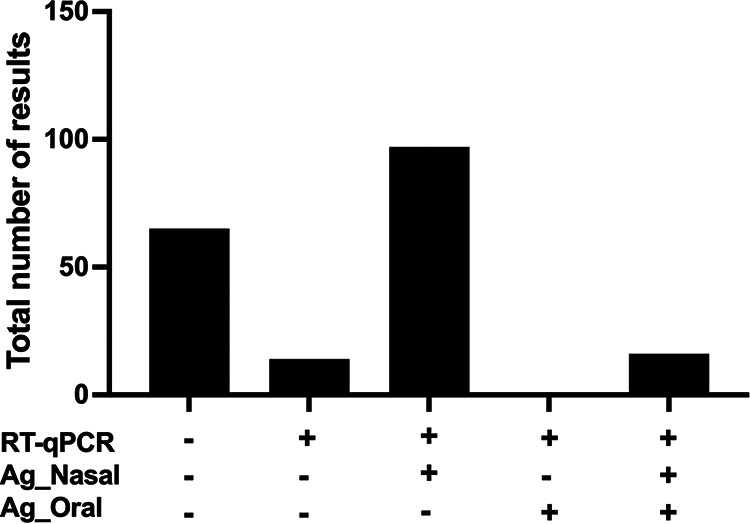
Concordance among SARS-CoV-2 RT-qPCR and Ag-RDT results from nasal (Ag_Nasal) and oral (Ag_Oral) specimens from 192 mildly symptomatic patients analyzed up to 7 days since symptom onset.

Analyzing the concordance among RT-qPCR and Ag-RDT results from different specimens, we obtained five possible results (Fig. 1). Most patients positive for SARS-CoV-2 RT-qPCR (*n* = 127) were also positive for nasal Ag-RDT (*n* = 113), while only 16 had positive Ag-RDT results from oral samples. The nasal Ag-RDT result showed the best concordance with RT-qPCR (*n* = 82), while Ag-RDT of oral samples alone failed to detect patients with positive SARS-CoV-2 RT-qPCR results (Fig. 1).

**FIG 2:**
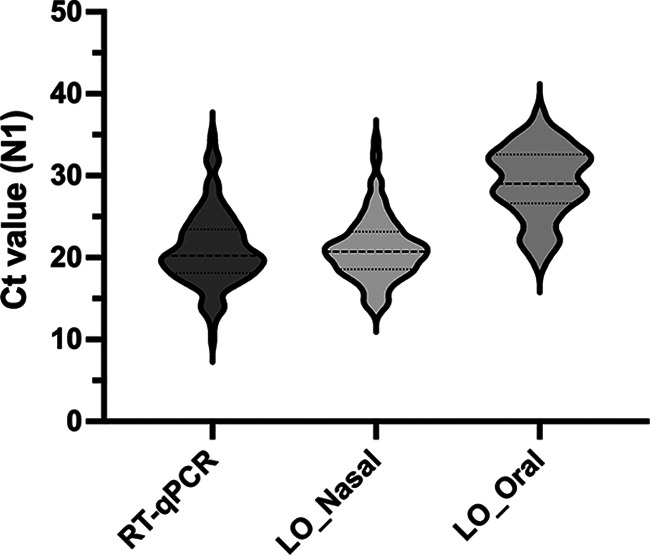
Violin plots showing the median, variability, and probability density of cycle threshold (Ct) values of N1 target amplification obtained by RT-qPCR from nasopharyngeal samples (RT-qPCR) and Ag-RDT leftovers (LO) from nasal and oral specimens in 192 mildly symptomatic patients tested up to 7 days since symptom onset. Medians were significantly different among groups (Kruskal-Wallis nonparametric test, *P* = 0.0009).

Median cycle threshold (*C_T_*) values of N1 target amplification by RT-qPCR of nasopharyngeal samples and Ag-RDT leftovers of nasal samples were in a similar range. However, oral Ag-RDT leftovers were significantly different (Fig. 2). The median *C_T_* values were higher in nasopharyngeal samples (*C_T_*, 19.48) and nasal (*C_T_*, 21.35) Ag-RDT leftovers than those obtained from oral (*C_T_*, 28.98) Ag-RDT leftovers (Kruskal-Wallis nonparametric test, *P* = 0.0009).

**FIG 3:**
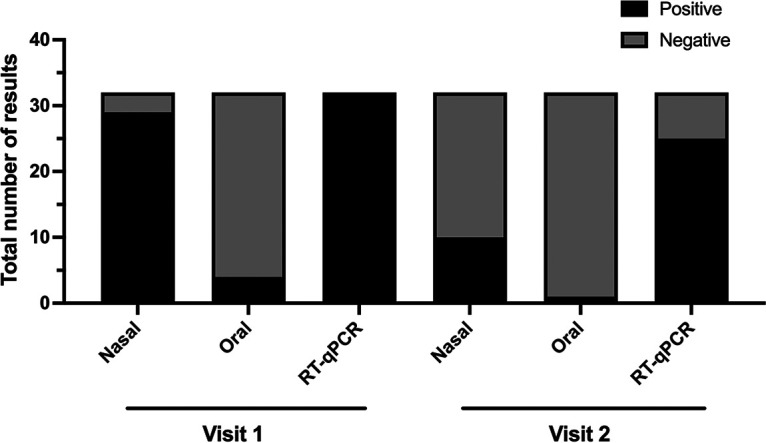
Total number of results obtained by RT-qPCR and Ag-RDT from nasal and oral specimens at the first and second visits in the follow-up group. A total of 32 patients with a positive SARS-CoV-2 RT-qPCR result were included for follow-up analysis until 7 days after the first test.

Ninety-seven specimens with positive SARS-CoV-2 RT-qPCR results and a *C_T_* value below 30 were segregated to run the VOC RT-qPCR. Thirty-two individuals with a positive SARS-CoV-2 RT-qPCR result had a second visit within 7 days, when Ag-RDTs of nasal, and oral specimens were tested again, as well as the RT-qPCRs of nasopharyngeal swabs (Fig. 3). At the second visit, the positivity rate of RT-qPCR fell to 78.1% (25/32), while the positivity rate of the Ag-RDT of nasal specimens fell to 31% (10/32) and that for the Ag-RDT of the oral specimens fell to 3% (Fig. 3).

**FIG 4:**
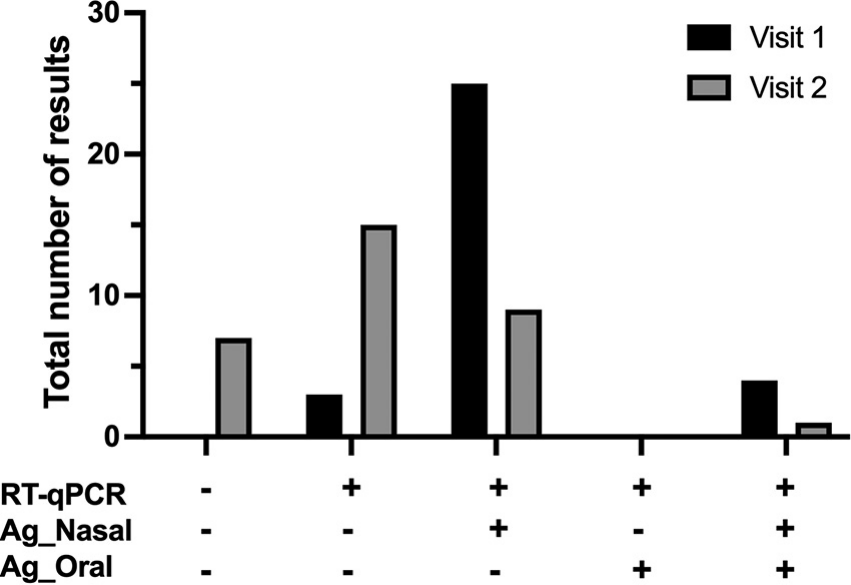
Concordance among SARS-CoV-2 RT-qPCR and Ag-RDT results from nasal (Ag_Nasal) and oral (Ag_Oral) specimens at the first and second visits in the follow-up group. A total of 32 patients with a positive SARS-CoV-2 RT-qPCR result were included for follow-up analysis until 7 days after the first test.

Figure 4 shows the concordance among RT-qPCR and Ag-RDTs results during initial and follow-up visits. The positive result observed with Ag-RDT of nasal specimens persisted longer than those detected from oral specimens. In fact, only nasal Ag-RDT maintained any concordance with the RT-qPCR result at the second visit, although the result was significantly lower than that observed at visit 1 (Fig. 4).

## DISCUSSION

Antigen rapid diagnostic tests (Ag-RDTs) are an important tool for point-of-care diagnosis of COVID-19, enabling the implementation of immediate control measures to avoid viral spread. The Panbio COVID-19 Ag rapid test device is an Ag-RDT for COVID-19 targeting the viral nucleocapsid protein (18, 19) and has been showing good performance against SARS-CoV-2 VOCs such as Alpha, Beta, Gamma, and Delta that circulated earlier during the SARS-CoV-2 pandemic (20).

The SARS-CoV-2 variant B.1.1.529 (Omicron accordingly to WHO nomenclature) originated in South Africa and was first reported on 24 November 2021 (6). The Omicron variant has 50 novel genomic mutations, of which 20 are in the S gene that encodes the spike protein (21). Omicron has a higher affinity for human ACE2 receptors than the Delta variant, indicating a higher potential for transmission (22, 23). Initial studies showed that infection by the Omicron variant produced less severe disease and significantly reduced the odds of hospital admission compared with earlier variants, such as Delta (24, 25). However, the onset of Omicron circulation raised concerns about Panbio COVID-19 Ag-RDT performance against this VOC as it incorporated mutations and deletions in the N protein that could potentially affect antigen binding. The Omicron VOC could also modify its cell tropism and infection kinetics, thus altering the site of viral replication throughout the disease course, thereby changing the appropriate specimen to collect for acute-phase diagnosis (8–12).

A study by Michelena et al., using nasopharyngeal samples (confirmed as Omicron variants by sequencing), showed that the Panbio COVID-I9 Ag rapid test device had high sensitivities—95.6% (*C_T_*, ≤20), 92.6% (*C_T_*, ≤25), 87.2% (*C_T_*, ≤30), and 81.8% (*C_T_*, ≤35)—compared with nasopharyngeal RT-PCR, with a specificity of 100% (26). In another study, Deerain et al. evaluated the analytical sensitivity of lateral flow devices against the SARS-CoV-2 Omicron variant using isolates cultured from clinical samples and demonstrated that the Panbio COVID-I9 Ag rapid test device consistently detected a sample (4/4 replicates [100%]) at a concentration of 6.39 log_10_ copies/mL, corresponding to a *C_T_* value of 25.8, underlining the high sensitivity of the test device (27).

A previous report suggested that the Omicron VOC has different replication sites in the initial phase of infection, leading to earlier detection in oral or saliva specimens instead of nasal ones (17). In this study, we examined the sensitivity and specificity of the Panbio COVID-19 Ag-RDT in 192 symptomatic individuals up to 7 days after symptom onset, using different specimens, including nasal bilateral middle-turbinate swabs and oral swab samples, compared with the gold standard RT-qPCR using nasopharyngeal swabs. The Panbio COVID-19 Ag-RDT showed good performance for Omicron detection when using nasal specimens, with 89% sensitivity and 100% specificity. However, oral specimens performed poorly, with only 12.6% sensitivity. It is noteworthy that Ag-RDT from oral specimens alone did not detect any patient with a positive SARS-CoV-2-RT-qPCR result, no matter how early tested after symptom onset. RT-qPCR from nasopharyngeal samples and nasal Ag-RDT showed positive results as early as 0 to 1 day after symptom onset.

Our data are in contrast with those reported by Marais et al. in 2021 (17), but support previous observations using the Panbio COVID-19 Ag RDT for other VOC detection (28). On the other hand, Lin et al. in 2022 reported no difference between nasal and oropharyngeal specimens for Omicron diagnosis when RT-PCR or Ag-RDT was tested (29). This discrepancy could potentially reflect our patient cohort, where most individuals were fully vaccinated with two doses of COVID-19 vaccine and a large number of patients had completed the third vaccine dose. This higher immune barrier could modify the natural initial replication site of the Omicron VOC in the oral cavity. Indeed, the poor performance of oral swabs in Ag-RDT in our samples could also be related to the fact that we only used oral and not oropharyngeal swabs and the nonoptimization of the sample buffer used for collection. Given saliva’s complex contents, optimization of the sample collection buffer would be crucial to increase Ag-RDT sensitivity (29).

We used leftover samples from the Panbio COVID-19 Ag RDT to perform RT-qPCR by a previously verified procedure originally set for nasopharyngeal as well as nasal swab collection (30). The amount of viral RNA, indicated by *C_T_* values obtained by RT-qPCR from the leftover samples, corroborated the Ag-RDT sensitivity using different specimens. These results showed lower viral concentration in the oral cavity than in the nasal cavity and nasopharynx, suggesting a virus load 32 times higher in nasal samples than in oral specimens. Furthermore, the follow-up of a subset of RT-qPCR-positive patients showed no variation in the viral load distribution among the upper respiratory tract compartments studied. Additionally, the *C_T_* values obtained in the second test corroborated the better performance of nasal specimens with Ag-RDT than oral specimens, supporting the finding that the nasal specimen is the best specimen type for acute COVID-19 diagnosis using the Panbio COVID-19 Ag-RDT. These results are consistent with the manufacturer’s instructions that require nasal or nasopharyngeal swab samples with the Panbio COVID-19 Ag RDT (18, 19). Studies using alternate specimen types, including oral swabs, not recommended in the product’s instructions for use, have produced lower sensitivity with the Panbio COVID-19 Ag RDT (31, 32).

To determine the genotype of the SARS-CoV-2 variant infecting our patient cohort, we used a VOC RT-qPCR assay that tests two sets of deletions (S106, G107, and F108 in the ORF1a gene coding for NSP6 and H69 and V70 in the spike gene) in SARS-CoV-2 genome. The results showed that 96 of the 97 tested specimens (99%) were infected with the Omicron VOC, confirming previous sequence studies in our laboratory showing complete replacement of the Delta VOC by Omicron in the community before sample collection for this study.

In conclusion, results from the present study demonstrated that Panbio COVID-19 Ag-RDT performance is unaffected in vaccinated individuals infected with the Omicron VOC. Furthermore, this study supported nasal specimens as the optimal specimen type for Panbio COVID-19 Ag-RDT performance compared with oral specimens.

## MATERIALS AND METHODS

### Study design and population.

This transversal study was conducted at the Center for COVID diagnosis at the Federal University of Rio de Janeiro. Subjects over 18 years old with mild COVID-19 suggestive symptoms, such as fever, cough, runny nose, sore throat, anosmia, ageusia, headache, diarrhea, and myalgia, for up to 7 days were tested. Samples were collected from 17 January to 7 February 2022.

### Sample collection.

A trained health care professional, and researcher in this study, collected four specimens from each participant. Samples included a nasal bilateral middle-turbinate swab, an oral swab, a saliva sample, and a nasopharyngeal swab. Nasal samples were collected according to the Panbio COVID-19 Ag test device instructions for use. The oral swab was collected as previously described (17). In summary, participants abstained from ingestion of food, drink, or chewing tobacco and gum for 30 min before oral sample collection. Participants coughed 3 to 5 times, covering their mouth with disposable tissue paper, before the operator swabbed the inside both cheeks, above and below the tongue, on gums, and on hard palate. After 5 to 10 min of rest, the operator collected a nasopharyngeal swab and immediately placed it in viral transport medium (VTM).

### Ag-RDT procedure.

Nasal and oral specimens were tested immediately following collection using the Panbio COVID-19 Ag test device (Abbott, Germany) according to the manufacturer's instructions. The manufacturer´s instructions point out the requirement for nasal or nasopharyngeal swab samples with the Panbio COVID-19 Ag RDT (18, 19). Tests were run and read within 15 min by trained technicians on-site in the testing center. The leftover samples from the Panbio elution tubes (~120 μL) were stored at 4°C and shipped to the laboratory for RT-qPCR within 4 h.

### Quantification of viral loads via RT-qPCR.

Viral RNA present in the leftover from the Panbio antigen tube after nasal and oral swab tests was extracted in a KingFisher Flex system (Thermo Fisher, USA), using the MagMax Viral/Pathogen kit (Thermo Fisher, USA). The SARS-CoV-2 (2019-nCoV) multiplex CDC qPCR probe assay (Integrated DNA Technologies, USA) targeting the SARS-CoV-2 N1 and N2 genes and the human RNase P (RNaseP) gene (endogenous control) detected viral RNA. A 7500 thermal cycler (Applied Biosystems, USA) was used for all reactions. RT-qPCR results were interpreted as positive if both targets (N1 and N2) were amplified with a cycle threshold (*C_T_*) value of ≤37.

VOC investigations were made on SARS-CoV-2 RT-qPCR-positive samples using a 4Plex SARS-CoV-2 for VOC screening kit (Bio-Manguinhos, Brazil). TaqMan probes for the SARS-CoV-2 virus were used for detection by amplifying a target region in the N gene and screening samples with suggestive profiles for the different VOCs. VOC profiles were given by combining results obtained of the deletions (Del) S106, G107, and F108 in the ORF1a gene (NSP6) and Del H69 and V70 in the spike gene from the samples tested. Samples were considered positive when *C_T_* values for SC2-N, Wt Del NSP6, and Wt Del 69, 70 were lower than 40.

### Statistical analysis.

The data from the study were acquired using the KoBoCollect online/offline web-based form (available at https://www.kobotoolbox.org). The data set was extracted on XLSForms and merged with the corresponding laboratory data. Ag-RDT sensitivity and specificity from different specimens were determined in relation to RT-qPCR. Sensitivity, specificity, predictive positive and negative values, as well as 95% confidence intervals (CI) were determined using Fisher's exact test. Differences among groups were assessed by Kruskal-Wallis nonparametric test. GraphPad Prism version 9.2.0 (GraphPad Software, San Diego, CA, USA) and JASP version 0.16 (JASP Team, 2021) were used. A *P* value of <0.05 was considered significant.

### Ethical statement.

The present study was approved by the local ethics review committee from Clementino Fraga Filho University Hospital (CAAE 30161620.0.0000.5257) and by the national ethical review board (CAAE 30127020.0.0000.0068). All enrolled participants signed written informed consent.
